# “Fading out” - genomic epidemiology of the last persistently infected BVDV cattle in Germany

**DOI:** 10.3389/fvets.2023.1339248

**Published:** 2024-01-04

**Authors:** Kerstin Wernike, Florian Pfaff, Martin Beer

**Affiliations:** Institute of Diagnostic Virology, Friedrich-Loeffler-Institut, Federal Research Institute for Animal Health, Greifswald - Insel Riems, Germany

**Keywords:** bovine viral diarrhea virus, pestivirus, persistent infection, control, sequence analysis, molecular typing, epidemiology, phylogeny

## Abstract

Bovine viral diarrhea virus (BVDV) is one of the most important cattle pathogens worldwide, causing major economic losses and animal welfare issues. Disease eradication programs have been implemented in several countries, including Germany where an obligatory nationwide control program is in force since 2011. As molecular epidemiology has become an essential tool to understand the transmission dynamics and evolution of BVDV, 5′ untranslated region (UTR) sequences are generated from viruses present in persistently infected animals since the beginning of the BVDV control program. Here, we report the results of the sequence-based subtyping of BVDV strains found from 2018 through 2022 in calves born in Germany. In 2018, 2019 and 2020, BVDV-1d and-1b were the dominant subtypes and cases were spread throughout the area that was not yet officially declared BVDV-free at that time. In addition, BVDV–1a, −1e, −1f and -1h could rarely be detected. From 2021 onwards, subtype 1d clearly took over the dominance, while the other subtypes could be gradually nearly eliminated from the cattle population. The eradication success not only results in a drastic reduction of cases, but also in a marked reduction of strain diversity. Interestingly, before vaccination has been banned in regions and farms with a disease-free status, two live-vaccine virus strains were repeatedly detected in ear tissue samples of newborn calves (*n* = 14) whose mothers were immunized during gestation. The field-virus sequences are an important basis for molecular tracing and identification of potential relationships between the last outbreaks in the final phase of the German BVDV eradication program, thereby supporting classic epidemiological investigations. Furthermore, the monitoring of the composition of virus subtypes in the cattle population helps to maintain effective diagnostic methods and control measures and is an early warning system for the introduction of new pestiviruses in the naïve cattle population.

## 1 Introduction

Bovine viral diarrhea virus (BVDV) is an economically important pathogen that affects cattle populations worldwide. It is a pestivirus within the *Flaviviridae* family, which also includes other important animal viruses such as classical swine fever virus (CSFV) and border disease virus (BDV) (1). BVDV is a single-stranded RNA virus that can be taxonomically divided into the virus species *Pestivirus bovis* (commonly known as BVDV-1), *Pestivirus tauri* (BVDV-2) and *Pestivirus brazilense* (BVDV-3 or HoBi-like pestivirus), which are further subdivided into subtypes based on genetic analysis (1, 2).

Clinical manifestations of bovine viral diarrhea (BVD) range from inapparent infections to acute diseases associated with diarrhea, fever, pneumonia, hemorrhagic lesions or the inevitably fatal mucosal disease (MD) (3). Additionally, BVDV can cause reproductive losses, immunosuppression, and predispose animals to secondary infections, leading to significant economic losses in the livestock industry. Infection during gestation often result in vertical virus transmission from the dam to the developing fetus, which, depending on the stage of pregnancy, may induce abortion, stillbirth, teratogenic effects or the birth of immunotolerant, persistently infected (PI), viremic calves (4, 5). PI animals are unable to develop a specific immune response to the specific BVDV strain they are infected with and therefore shed huge amounts of virus throughout their lives. Hence, they are the major and epidemiologically most relevant source for virus transmission and maintenance in cattle populations (6–9). Another major cause for BVDV transmission to hitherto unaffected farms are so-called “Trojan cows,” i.e., dams infected during early gestation and therefore pregnant with a PI fetus (7, 8, 10–12). Such “Trojan cows” are particularly challenging for the diagnosis and control of the disease, as the problem becomes only visible when the PI calf is born, which could be several months after the actual infection of the dam.

To counteract the significant economic and animal welfare consequences of BVD, control programs have been implemented in several countries (13, 14). The common goal is the elimination of BVDV by the detection and removing of PI animals as early as possible from the respective cattle population, however, there are two different approaches to achieve this goal.

The so-called “Scandinavian model” is based on large-scale bulk milk serology combined with a vaccination ban to preselect farms with a higher risk for the presence of PI animals. Thereafter, all animals from these herds are tested individually to identify and remove the PI animal(s). In contrast, the “Swiss approach” is based on the direct viral genome or antigen testing of all animals without a serological pre-screening of the cattle herds. This approach proved beneficial for countries with high initial BVDV prevalences, high levels of cattle trade and when vaccination is permitted. The latter approach has also been chosen for Germany, where BVD/MD has been a notifiable disease since 2004 and an obligatory nationwide eradication program was started in 2011 (15). The defined basis rules are the mandatory testing of each newborn calf for BVDV antigen or genome leading to nearly 5 million diagnostic BVDV tests every year (15), the immediate elimination of all detected virus-positive animals, and trade with certified unsuspicious animals only. From 2011 to May 2016, the calves had to be investigated in the first 6 months of life and from June 2016 to March 2021 in the first 4 weeks. Since April 2021, when the Animal Health Law (AHL) including its accompanying delegated regulations came into force in the European Union (EU), every calf needs to be tested in the first 20 days post-partum. Vaccination was permitted at the beginning of the program, but has been banned since 2021 in regions with a disease-free status or with an approved eradication program according to EU law. Several inactivated vaccines (monovalent preparations or in combination with immunogens against further pathogens) and the two live vaccines Bovela® (Boehringer Ingelheim Vetmedica GmbH, Ingelheim/Rhein, Germany) and Vacoviron® FS (Merial GmbH, Hallbergmoos, Germany) were applied. Bovela® is based on the virus strains KE-9 (subtype BVDV-1b) and NY-93 (subtype BVDV-2a) and Vacoviron® includes the BVDV-1a vaccine virus Oregon C24V, which has been used since the 1960s in Europe (16, 17).

Since the onset of the obligatory German BVD control program in 2011 until 2022, the proportion of animals classified as PI among all newborn calves could be reduced with each passing year, starting with 0.5% in 2011 and resulting in 0.001% (55 PI calves among about 4.3 Mio. newborn calves) in 2022 (18).

The highly successful reduction of newly born PI animals also led to a drastic reduction of BVDV sequences. As genomic epidemiology has become an important tool for understanding the transmission dynamics and evolution of BVDV (19), a comprehensive sequence data set of the 5′ untranslated region (UTR) of the viral genome has been generated during the early phases of the German control program. In this context, a considerable variety of BVDV subtypes had been identified (20). Here, we present the follow-up, i.e., the results of the 5′UTR sequence-based subtyping of the remaining virus strains found in the most recent German BVD cases. The sequences are used for molecular tracing of the last outbreaks in the final phase of the country’s eradication program, as the typing of viral isolates proved beneficial for the identification of relationships between BVD outbreaks, thereby identifying risk factors for virus transmission.

## 2 Materials and methods

In Germany, every newborn calf is tested for BVDV by either real-time RT-PCR or antigen ELISA. The tests are carried out in the regional laboratories and most of them submit BVDV-positive samples to the national reference laboratory (NRL) for BVD/MD for further characterization of the detected viruses. The real-time RT-PCRs used in the regional laboratories allow for the detection of the three BVDV species (21, 22). The diagnostic samples submitted to the NRL are either viral RNA or blood samples or ear notch samples soaked in antigen-ELISA buffer or direct lysis buffer. In very rare cases, nasal swabs are submitted. Here, we describe the genetic characterization of the BVDV strains that were found in PI animals born between 2018 and 2022.

From the submitted blood samples and swab fluids, viral RNA was extracted using the QIAamp Viral RNA Mini Kit (Qiagen GmbH, Hilden, Germany) according to the manufacturer’s recommendation. The ear notch samples were manually recovered from the original tubes containing antigen-ELISA or direct lysis buffer, homogenized in RLT buffer (Qiagen, Hilden, Germany), and subsequently viral RNA was extracted using the RNeasy Mini Kit (Qiagen, Hilden, Germany). The presence of pestivirus genome was confirmed by a previously published generic panpesti real-time RT-PCR (20). For real-time RT-PCR positive samples, sequence information of the 5′UTR was generated as described previously in both directions using the primers BVD I (5′-GGT AGC AAC AGT GGT GAG TTC-3′) and UTR51 (5′-CAA CTC CAT GTG CCA TGT AC-3′) (258 base pairs) or BVD II (5′-AGC GGT AGC AGT GAG TTC ATT-3′) and UTR51 (5′-CAA CTC CAT GTG CCA TGT AC-3′) (257 base pairs) (23). The sequences were assembled and aligned using Geneious Prime version 2021.0.1 (Biomatters, Auckland, New Zealand). The sequences generated in this study were submitted to the International Nucleotide Sequence Database Collaboration databases (GenBank accession numbers OR710303 to OR710491).^1^

To determine the BVDV subtypes, sequences of representative reference strains available at NCBI GenBank were used for comparison. In cases of the detection of the modified live-vaccine strain KE-9, the double individual genomic deletions were confirmed by Sanger sequencing as described previously (20). When the genomic deletions were confirmed or when another live-vaccine strain was identified by 5′UTR sequencing, the samples were excluded from analyses of the spatial distribution of BVDV subtypes. When multiple samples from individual cattle holding were submitted to the NRL and the 5′UTR sequences generated from these samples were identical, only the first PI animal within a given year was included in further analyses for that year. Maximum-likelihood trees were calculated separately for each year from 2018 to 2022 using the Kimura 2-parameter model (24) with 1,000 bootstrap replicates by using the MEGA X software (25).

For illustration of the spatial distribution of the BVDV subtypes, the districts of birth of the calves were either retrieved from the German cattle database (HI-Tier) based on the ear tag number of the animals or they were indicated in the letter accompanying the samples.

## 3 Results

The live-vaccine strains Oregon C24V (included in, e.g., the vaccines Vacoviron® FS [Merial GmbH, Hallbergmoos, Germany], and MUCOSIFFA [Ceva Santé Animale, Libourne, France]), and KE-9 (Bovela® [Boehringer Ingelheim Vetmedica GmbH, Ingelheim/Rhein, Germany]) were found in ear notch samples of seven newborn calves each. The numbers per year are provided in Table 1. Those samples were excluded from the analyses of the spatial distribution of circulating BVDV strains.

**Table 1:** Number of detections of live-vaccine strains Oregon C24V and KE-9 in newborn calves after vaccination of their mothers in early pregnancy.

year	Oregon C24V	KE-9
2018	2	2
2019	3	0
2020	1	3
2021	1	2
2022	0	0
total	7	7

**Figure 1:**
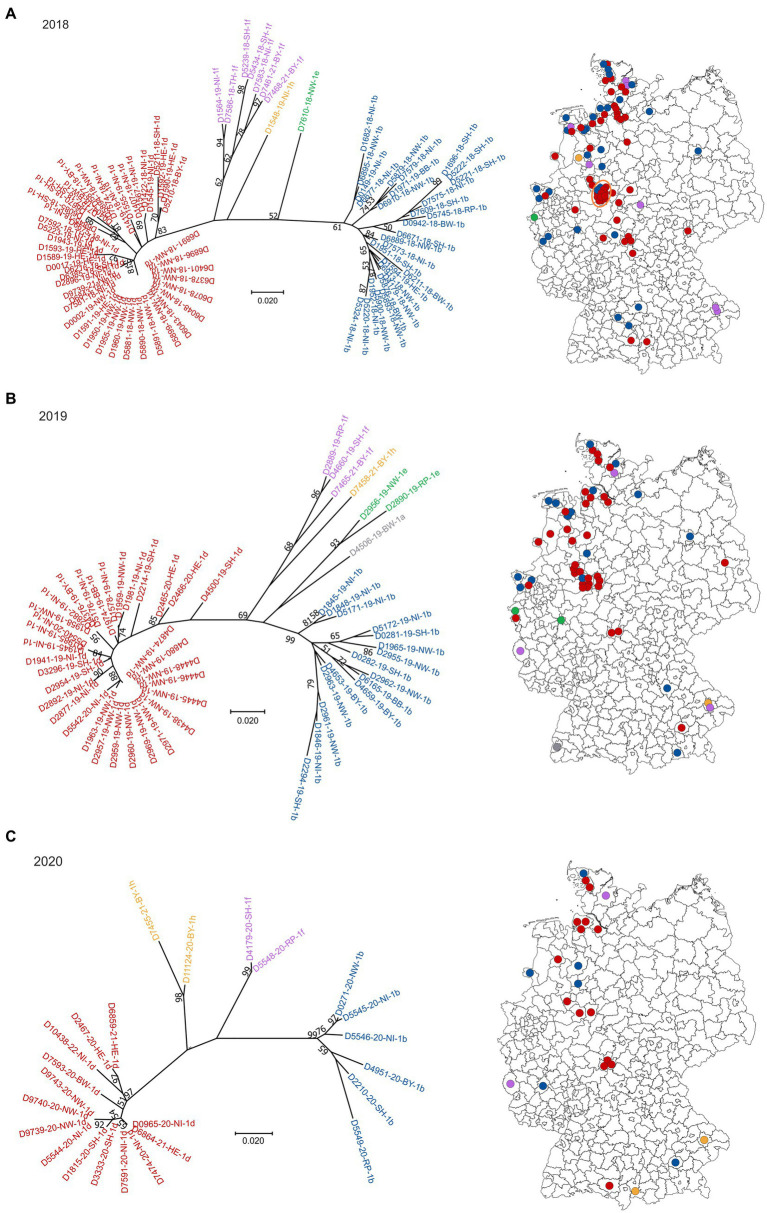

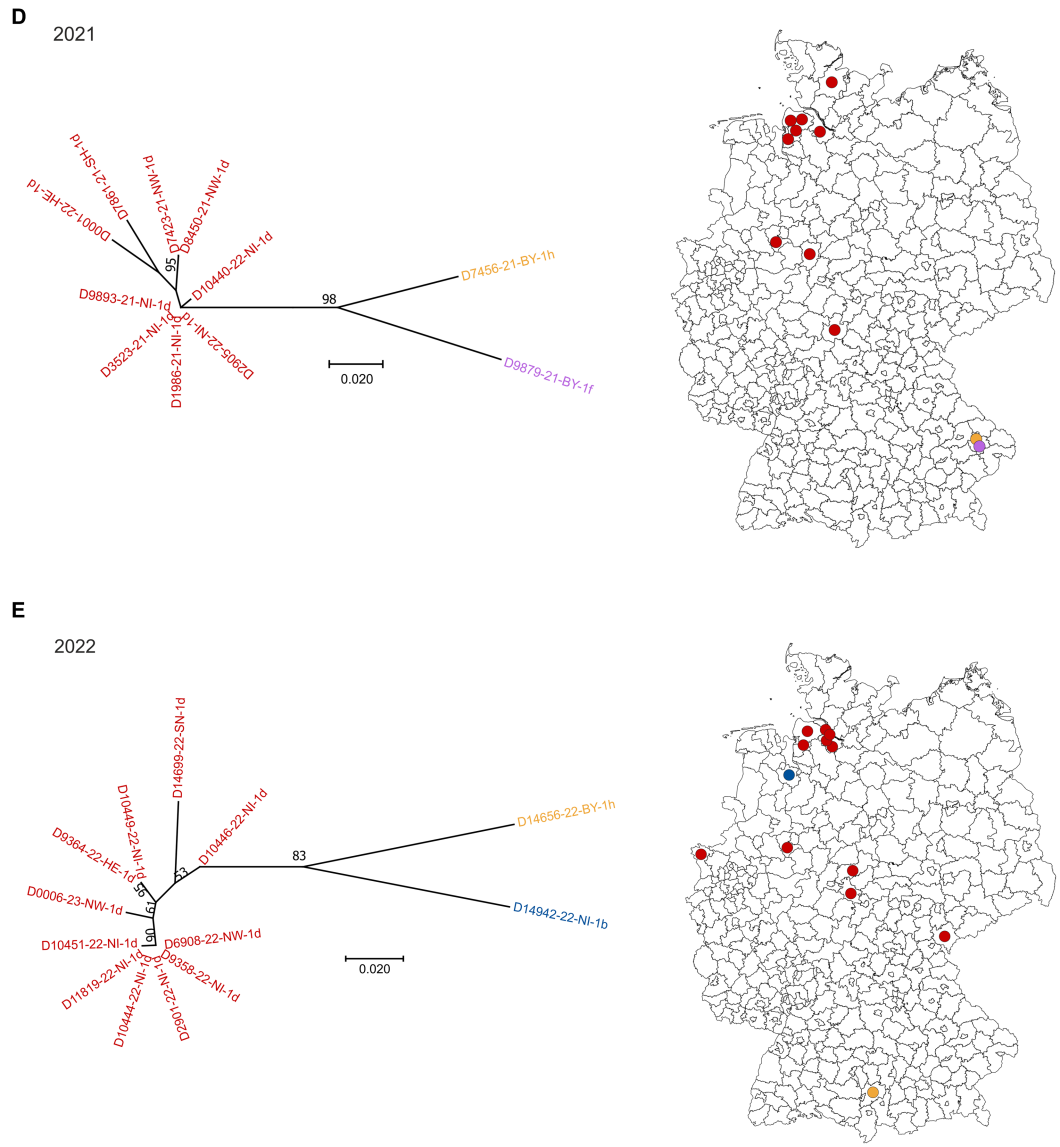
BVDV subtypes detected in PI calves in Germany in 2018 **(A)**, 2019 **(B)**, 2020 **(C)**, 2021 **(D)** and 2022 **(E)**. On the left side of each panel, the phylogenetic classification based on the 5’UTR is depicted. Strains that belong to BVDV subtype 1a are shown grey, 1b in blue, 1d in red, 1e in green, 1f in purple and 1h in yellow. The distribution of the subtypes within Germany (right) is shown using the identical colors. Dots are randomly distributed in the districts, meaning that the exact location of the farms within the respective district cannot be deduced. The orange circle in panel **(A)** indicates a district with a particular high number of BVDV-infected cattle farms. The map basis, in which the dots were printed, was retrieved from: Bundesamt für Kartographie und Geodäsie (2022), data sources: Statistisches Bundesamt (Destatis), Bundesinstitut für Bau-, Stadt-und Raumforschung (BBSR), Data licence Germany – attribution – Version 2.0 (www.govdata.de/dl-de/by-2-0).

Sequences of field-viruses could be generated from PI animals born in 85 (year 2018), 56 (year 2019), 24 (year 2020), 11 (year 2021) and 13 (year 2022) cattle farms, respectively, resulting in a dataset of 189 sequences of the 5′UTR genomic region (Figure 1). The annual drop in the number of BVDV-positive samples reflects the overall decline in PI herds in Germany due to the efficient eradication process (18). The number of cattle holdings from which BVDV sequences were generated account for about 68% of all PI herds in 2018, 60% in 2019, 49% in 2020, 42% in 2021 and 54% in 2022. The spatial distribution of the sequenced samples (Figure 1) reflect that of the reported BVD cases, with the presence of PI animals predominantly in Western and Southern Germany (18).

In 2018, 2019 and 2020, BVDV-1d and-1b were the dominant subtypes and cases were spread throughout the area that was officially classified as “not BVD-free” at that time (Figures 1A–C). Subtype 1a was detected only once in 2019, subtype 1e once in 2018 and two times in 2019, and subtypes 1f and 1h were found a few times throughout the years. From the year 2021 onwards, subtype 1d was clearly dominant, as all but two sequences belonged to this virus type in 2021 and 2022, respectively (Figures 1D,E). In the two remaining cases of both years, BVDV of the subtypes 1f and 1h (2021) or 1b and 1h (2022) were found.

When analyzing the spatial distribution in 2018, a district in central-western Germany stands out, where multiple farms were affected (orange circle in Figure 1A). In these cases, all but two viral 5′UTR sequences belonged to subtype BVDV-1d and all of them were identical, indicating very clearly a common source of infection or an infection chain between the respective farms. In the following year, a similar distribution was seen, but fewer farms were affected and the virus strain was additionally found in the neighboring district. In 2020, however, the larger outbreak has ended, and only one viral 5′UTR sequence was generated per district. In both cases, the sequences belonged to subtype BVDV-1d and were identical to those of the preceding years.

From samples collected in four cattle holdings in 2018/2019 whole genome sequences were generated previously (26). The comparison of these sequences revealed very high nucleotide identity between the individual samples (>99.6%) (26), further supporting the assumption that there was either a common source of infection or an infection chain between the respective farms in this district.

## 4 Discussion

Understanding the diversity of BVDV strains in a given area is essential to maintain effective diagnostic tools and control measures. When control strategies include vaccination or vaccination is applied in areas without systematic BVD control, one needs to keep in mind that BVD vaccination is complicated by the wide antigenic diversity among field viruses (27). BVDV exists in three species with numerous subtypes (1, 2) and this diversity poses challenges in vaccine efficacy, which can vary depending on the specific virus subtypes. Hence, it is important to monitor which virus types are circulating in order to select the most suitable vaccine to match the prevalent strains in a given region. Another aspect to consider when implementing BVD immunization strategies is the type of vaccine. Several vaccine formulations have been developed against BVDV infections, ranging from classical inactivated to subunit, modified live virus (MLV), chimeric pestiviruses, replicons and synthetic attenuated infectious cDNA clones (28). All of them have their specific advantages and disadvantages. While inactivated vaccines are considered risk-free at any age or phase of gestation, they are less efficient, booster vaccinations are required in most cases and the extent of fetal protection is not clear (29). On the other hand, MLV vaccines often induce a broad, robust and longer-lasting immune response (30, 31), but may pose a risk of vertical transmission through the placental barrier from the dam to the developing fetus. When live-vaccine strains persist for an extended period within fetal tissues, possibly until after birth, they may be detectable in the newborns, as has been reported earlier (20, 32) and was again observed repeatedly in our study. Considering the successful progress of the German BVD control program (18) and based on a 6-month follow-up of a calf in whose ear tissue the commercial Npro and Erns double mutant BVDV-1 live-vaccine strain KE-9 was found (32), the epidemiological impact of the RNA/antigen positivity in the skin of newborns appears to be very low. However, it presents a diagnostic issue, which may lead to culling measures when sequence analysis to differentiate vaccine strains from field-viruses is not performed. Therefore, careful consideration should be given to whether pregnant animals are immunized with MLV vaccines.

Among the field viruses, BVDV subtypes 1d and 1b were the most prevalent ones and subtypes 1a, 1e, 1f and 1h were additionally detected, all of them belonging to the species *Pestivirus bovis*. Interestingly, neither BVDV-2, which caused in major disease outbreak in Western Germany in 2012/13 (33), nor BVDV-3 (HoBi-like pestivirus) were found between 2018 and 2022. BVDV-3 was originally isolated from a Brazilian fetal calf serum (34) and has since been found predominantly in further South American countries and Asia, but also in Italy (35, 36). But fortunately, it appears that BVDV-3 has not been yet introduced into the German cattle population and spread to a larger extent, as it was not detected in any of the samples analyzed in this study. Nevertheless, monitoring and typing of circulating viruses should be maintained in order to detect any novel introduction of diverse virus variants or even other pestivirus species into the naïve cattle population as early as possible.

Besides the possibility to notice introductions of non-endemic or even novel pestiviruses, genomic epidemiology enables a monitoring of circulating strains, thereby supporting classic epidemiological outbreak investigations. This is especially beneficial in the case of BVD, where several months may pass between the infection of the dam and the time point when the problem becomes visible, i.e., when a PI calf is born. Consequently, such molecular-epidemiological approaches have been implemented in numerous countries (19, 37–42). A limitation of our study might be that only 5’UTR sequences were used. Supplementary sequencing of further genome regions (e.g., N^pro^) or even whole genome sequencing could potentially increase accuracy by providing more comprehensive genomic information. However, partial 5’UTR sequencing offers several advantages over whole genome sequencing, primarily cost-effectiveness and low turn-around time. Besides, 5’UTR sequencing is more commonly used than, e.g., N^pro^ sequencing (19, 40, 41) and, therefore, allows for easy comparison with existing databases and published sequences, aiding in strain identification and subtyping. Most importantly, the 5’UTR region of BVDV contains sufficient genetic variation to differentiate viral subtypes. Here, we identified the viruses from the infection hotspot in central-western Germany as belonging to BVDV subtype 1d, which agrees with whole genome-based subtyping of a subset of the samples from this outbreak series (26).

The high genetic identity of viruses detected in multiple PI animals in several farms hint at a common source of infection or infection chains between the affected cattle holdings. Indeed, additional epidemiological investigations have identified a common source that could be eliminated, thereby bringing an end to the outbreak series in combination with systematic testing, immediate elimination of each detected PI animal and strict biosecurity measures. From a national perspective, the combination of testing, removing PI animals and biosecurity did not just lead to the eradication of individual infection hotspots, but to a marked overall drop in the births of PI animals related to the total number of newborn calves (18) up to a disease-free status of multiple federal states. In this context, the number of detections of certain subtypes (e.g., BVDV-1b) decreased markedly and some virus subtypes (e.g., BVDV-1a and 1e) could even be eliminated from the cattle population, thereby reducing the genetic diversity to a minimum. Nonetheless, there is a constant risk of re-introduction by, e.g., trade with untested calves, import of cattle with uncertain BVD status or purchase of “Trojan cows.” Therefore, it is essential to sustain monitoring programs also in disease-free regions to maintain the BVDV-free status. Since one result of a consequent eradication and vaccination ban is BVDV antibody naïve cattle populations, the risk of infections with border disease virus from sheep - as shown previously, e.g., in Switzerland (43) - should be especially taken into consideration.

In conclusion, the German nationwide BVD control program led to a highly efficient reduction of PI animals and as a side effect to the gradual elimination of certain BVDV subtypes from the cattle population. Virus subtyping and molecular-epidemiological investigations can assist in the identification of transmission chains, thereby supporting the prevention of virus transmission to hitherto unaffected farms and aiding in the maintenance of control measures.

## Data availability statement

The datasets presented in this study can be found in online repositories. The names of the repository/repositories and accession number(s) can be found at: https://www.ncbi.nlm.nih.gov/genbank/, OR710303 to OR710491.

## Ethics statement

Ethical approval was not required for the study involving animals in accordance with the local legislation and institutional requirements because the animal samples were taken by the responsible farm veterinarians in the context of the German mandatory BVD control program and as prescribed in the BVDV regulation (BVDV-Verordnung in der Fassung der Bekanntmachung vom 27. Juni 2016, BGBl. I S. 1483).

## Author contributions

KW: Conceptualization, Formal analysis, Investigation, Supervision, Visualization, Writing – original draft. FP: Formal analysis, Investigation, Writing – review & editing. MB: Conceptualization, Supervision, Writing – review & editing.

## Footnotes

^1^ www.insdc.org

## References

[1] ICTV. Family: *Flaviviridae*, genus: *Pestivirus*. Available at: https://talkictvonlineorg/ictv-reports/ictv_online_report/positive-sense-rna-viruses/w/flaviviridae/361/genus-pestivirus. (2023).

[2] Vilcek S Paton DJ Durkovic B Strojny L Ibata G Moussa A. Bovine viral diarrhoea virus genotype 1 can be separated into at least eleven genetic groups. Arch Virol. (2001) 146:99–115. doi: 10.1007/s007050170194, PMID: 11266221

[3] Lanyon SR Hill FI Reichel MP Brownlie J. Bovine viral diarrhoea: pathogenesis and diagnosis. Vet J. (2014) 199:201–9. doi: 10.1016/j.tvjl.2013.07.024 24053990

[4] Baker JC. The clinical manifestations of bovine viral diarrhea infection. Vet Clin North Am Food Anim Pract. (1995) 11:425–45. doi: 10.1016/S0749-0720(15)30460-6 8581856

[5] Brock KV. The persistence of bovine viral diarrhea virus. Biologicals. (2003) 31:133–5. doi: 10.1016/S1045-1056(03)00029-0 12770545

[6] Ezanno P Fourichon C Seegers H. Influence of herd structure and type of virus introduction on the spread of bovine viral diarrhoea virus (BVDV) within a dairy herd. Vet Res. (2008) 39:39. doi: 10.1051/vetres:2008016, PMID: 18346451

[7] Akagami M Seki S Kashima Y Yamashita K Oya S Fujii Y. Risk factors associated with the within-farm transmission of bovine viral diarrhea virus and the incidence of persistently infected cattle on dairy farms from Ibaraki prefecture of Japan. Res Vet Sci. (2020) 129:187–92. doi: 10.1016/j.rvsc.2020.02.001, PMID: 32078846

[8] Bitsch V Hansen KE Ronsholt L. Experiences from the Danish programme for eradication of bovine virus diarrhoea (BVD) 1994–1998 with special reference to legislation and causes of infection. Vet Microbiol. (2000) 77:137–43. doi: 10.1016/S0378-1135(00)00270-4, PMID: 11042407

[9] Tråvén M Alenius S Fossum C Larsson B. Primary bovine viral diarrhoea virus infection in calves following direct contact with a persistently viraemic calf. Zentralbl Veterinarmed B. (1991) 38:453–62. doi: 10.1111/j.1439-0450.1991.tb00895.x 1719713

[10] Reardon F Graham DA Clegg TA Tratalos JA O'Sullivan P More SJ. Quantifying the role of Trojan dams in the between-herd spread of bovine viral diarrhoea virus (BVDv) in Ireland. Prev Vet Med. (2018) 152:65–73. doi: 10.1016/j.prevetmed.2018.02.002, PMID: 29559107

[11] Albrecht K Linder M Heinrich A Höche J Beer M Gaede W. Re-introduction of bovine viral diarrhea virus in a disease-free region: Impact on the affected cattle herd and diagnostic implications. Pathogens. (2021) 10:360. doi: 10.3390/pathogens10030360 33803542 PMC8002923

[12] Van Duijn L Santman-Berends I Biesheuvel M Mars J Waldeck F van Schaik G. Why test purchased cattle in BVDV control programs? Front vet sci. (2021) 8:686257. doi: 10.3389/fvets.2021.686257, PMID: 34513967 PMC8429825

[13] Moennig V Becher P. Control of bovine viral diarrhea. Pathogens. (2018) 7:29. doi: 10.3390/pathogens7010029 29518049 PMC5874755

[14] Stahl K Alenius S. BVDV control and eradication in Europe - an update. Jpn J Vet Res. (2012) 60:S31–9. 22458198

[15] Wernike K Gethmann J Schirrmeier H Schröder R Conraths FJ Beer M. Six years (2011-2016) of mandatory nationwide bovine viral diarrhea control in Germany - a success story. Pathogens. (2017) 6:50. doi: 10.3390/pathogens6040050 29057796 PMC5750574

[16] Coggins L Gillespie JH Robson DS Thompson JD Phillips WV Wagner WC. Attenuation of virus diarrhea virus (strain Oregon C24V) for vaccine purposes. Cornell Vet. (1961) 51:539–45. 13880178

[17] Balint A Baule C Palfi V Belak S. Retrospective genome analysis of a live vaccine strain of bovine viral diarrhea virus. Vet Res. (2005) 36:89–99. doi: 10.1051/vetres:2004053, PMID: 15610726

[18] Friedrich-Loeffler-Institut. Statistik zur BVD-Bekämpfung in Deutschland: PI-Tiere (Zeitraum 2011–2022). Available at: https://wwwflide/de/institute/institut-fuer-virusdiagnostik-ivd/referenzlabore/nrl-fuer-bvdmd/. (2023).

[19] Stalder H Hug C Zanoni R Vogt HR Peterhans E Schweizer M. A nationwide database linking information on the hosts with sequence data of their virus strains: a useful tool for the eradication of bovine viral diarrhea (BVD) in Switzerland. Virus Res. (2016) 218:49–56. doi: 10.1016/j.virusres.2015.09.012, PMID: 26403669

[20] Wernike K Schirrmeier H Strebelow HG Beer M. Eradication of bovine viral diarrhea virus in Germany-diversity of subtypes and detection of live-vaccine viruses. Vet Microbiol. (2017) 208:25–9. doi: 10.1016/j.vetmic.2017.07.009, PMID: 28888645

[21] Wernike K Beer M. Diagnostics in the context of an eradication program: results of the German bovine viral diarrhea proficiency trial. Vet Microbiol. (2019) 239:108452. doi: 10.1016/j.vetmic.2019.108452, PMID: 31767099

[22] Hoffmann B Depner K Schirrmeier H Beer M. A universal heterologous internal control system for duplex real-time RT-PCR assays used in a detection system for pestiviruses. J Virol Methods. (2006) 136:200–9. doi: 10.1016/j.jviromet.2006.05.020, PMID: 16806503

[23] Schaarschmidt U Schirrmeier H Strebelow G Wolf G. Detection of border disease virus in a sheep flock in Saxonia. Berl Munch Tierarztl. (2000) 113:284–8. 10994254

[24] Kimura M. A simple method for estimating evolutionary rates of base substitutions through comparative studies of nucleotide sequences. J Mol Evol. (1980) 16:111–20. doi: 10.1007/BF01731581, PMID: 7463489

[25] Kumar S Stecher G Li M Knyaz C Tamura K. MEGA X: molecular evolutionary genetics analysis across computing platforms. Mol Biol Evol. (2018) 35:1547–9. doi: 10.1093/molbev/msy096, PMID: 29722887 PMC5967553

[26] King J Pohlmann A Dziadek K Beer M Wernike K. Cattle connection: molecular epidemiology of BVDV outbreaks via rapid nanopore whole-genome sequencing of clinical samples. BMC Vet Res. (2021) 17:242. doi: 10.1186/s12917-021-02945-3, PMID: 34247601 PMC8272987

[27] Moennig V Becher P. Pestivirus control programs: how far have we come and where are we going? Anim Health Res Rev. (2015) 16:83–7. doi: 10.1017/S1466252315000092, PMID: 26050577

[28] Riitho V Strong R Larska M Graham SP Steinbach F. Bovine pestivirus heterogeneity and its potential impact on vaccination and diagnosis. Viruses. (2020) 12:1134. doi: 10.3390/v12101134, PMID: 33036281 PMC7601184

[29] Moennig V Eicken K Flebbe U Frey HR Grummer B Haas L. Implementation of two-step vaccination in the control of bovine viral diarrhoea (BVD). Prev Vet Med. (2005) 72:109–14. doi: 10.1016/j.prevetmed.2005.08.011, PMID: 16169620

[30] Griebel PJ. BVDV vaccination in North America: risks versus benefits. Anim Health Res Rev. (2015) 16:27–32. doi: 10.1017/S1466252315000080, PMID: 26050569

[31] Plotkin SA. Vaccines: the fourth century. Clin Vaccine Immunol. (2009) 16:1709–19. doi: 10.1128/CVI.00290-09, PMID: 19793898 PMC2786381

[32] Wernike K Michelitsch A Aebischer A Schaarschmidt U Konrath A Nieper H. The occurrence of a commercial N(pro) and E(rns) double mutant BVDV-1 live-vaccine strain in newborn calves. Viruses. (2018) 10:274. doi: 10.3390/v10050274, PMID: 29783722 PMC5977267

[33] Gethmann J Homeier T Holsteg M Schirrmeier H Sasserath M Hoffmann B. BVD-2 outbreak leads to high losses in cattle farms in Western Germany. Heliyon. (2015) 1:e00019. doi: 10.1016/j.heliyon.2015.e00019, PMID: 27441213 PMC4939757

[34] Schirrmeier H Strebelow G Depner K Hoffmann B Beer M. Genetic and antigenic characterization of an atypical pestivirus isolate, a putative member of a novel pestivirus species. J Gen Virol. (2004) 85:3647–52. doi: 10.1099/vir.0.80238-0, PMID: 15557237

[35] Bauermann FV Ridpath JF. HoBi-like viruses--the typical 'atypical bovine pestivirus. Anim Health Res Rev. (2015) 16:64–9. doi: 10.1017/S146625231500002X, PMID: 26050574

[36] Decaro N Lucente MS Mari V Sciarretta R Pinto P Buonavoglia D. Hobi-like pestivirus in aborted bovine fetuses. J Clin Microbiol. (2012) 50:509–12. doi: 10.1128/JCM.05887-11, PMID: 22162547 PMC3264192

[37] Luzzago C Decaro N. Epidemiology of bovine pestiviruses circulating in Italy. Front vet sci. (2021) 8:669942. doi: 10.3389/fvets.2021.669942, PMID: 34150891 PMC8206264

[38] Schoepf K Revilla-Fernandez S Steinrigl A Fuchs R Sailer A Weikel J. Retrospective epidemiological evaluation of molecular and animal husbandry data within the bovine viral diarrhoea virus (BVDV) control programme in Western Austria during 2009-2014. Berl Munch Tierarztl. (2016) 129:196–201. doi: 10.2376/0005-9366-129-15102 27344911

[39] Vilcek S Rossmanith W. The role of molecular-genetic techniques in BVDV eradication in Lower Austria. Vet Ital. (2022) 58. doi: 10.12834/VetIt.2595.16049.1 37303140

[40] Monteiro FL Martins B Cargnelutti JF Noll JG Weiblen R Flores EF. Genetic identification of pestiviruses from beef cattle in southern Brazil. Braz J Microbiol. (2019) 50:557–63. doi: 10.1007/s42770-019-00058-6, PMID: 30877664 PMC6863326

[41] Wang L Wu X Wang C Song C Bao J Du J. Origin and transmission of bovine viral diarrhea virus type 1 in China revealed by phylodynamic analysis. Res Vet Sci. (2019) 128:162–9. doi: 10.1016/j.rvsc.2019.11.015 31809973

[42] Booth RE Thomas CJ El-Attar LM Gunn G Brownlie J. A phylogenetic analysis of bovine viral Diarrhoea virus (BVDV) isolates from six different regions of the UK and links to animal movement data. Vet Res. (2013) 44:43. doi: 10.1186/1297-9716-44-43, PMID: 23783173 PMC3691640

[43] Braun U Hilbe M Janett F Hässig M Zanoni R Frei S. Transmission of border disease virus from a persistently infected calf to seronegative heifers in early pregnancy. BMC Vet Res. (2015) 11:43. doi: 10.1186/s12917-014-0275-7, PMID: 25889936 PMC4336514

